# Analysis of the role of Ser1/Ser2/Thr9 phosphorylation on myosin II assembly and function in live cells

**DOI:** 10.1186/1471-2121-12-52

**Published:** 2011-12-02

**Authors:** Jordan R Beach, Lucila S Licate, James F Crish, Thomas T Egelhoff

**Affiliations:** 1 Department of Cell Biology, Lerner Research Institute NC-10, Cleveland Clinic, 9500 Euclid Avenue, Cleveland, OH 44195, USA; 2 Department of Physiology and Biophysics, Case Western Reserve University, 10900 Euclid Avenue, Cleveland, OH 44106, USA

## Abstract

**Background:**

Phosphorylation of non-muscle myosin II regulatory light chain (RLC) at Thr18/Ser19 is well established as a key regulatory event that controls myosin II assembly and activation, both in vitro and in living cells. RLC can also be phosphorylated at Ser1/Ser2/Thr9 by protein kinase C (PKC). Biophysical studies show that phosphorylation at these sites leads to an increase in the Km of myosin light chain kinase (MLCK) for RLC, thereby indirectly inhibiting myosin II activity. Despite unequivocal evidence that PKC phosphorylation at Ser1/Ser2/Thr9 can regulate myosin II function in vitro, there is little evidence that this mechanism regulates myosin II function in live cells.

**Results:**

The purpose of these studies was to investigate the role of Ser1/Ser2/Thr9 phosphorylation in live cells. To do this we utilized phospho-specific antibodies and created GFP-tagged RLC reporters with phosphomimetic aspartic acid substitutions or unphosphorylatable alanine substitutions at the putative inhibitory sites or the previously characterized activation sites. Cell lines stably expressing the RLC-GFP constructs were assayed for myosin recruitment during cell division, the ability to complete cell division, and myosin assembly levels under resting or spreading conditions. Our data shows that manipulation of the activation sites (Thr18/Ser19) significantly alters myosin II function in a number of these assays while manipulation of the putative inhibitory sites (Ser1/Ser2/Thr9) does not.

**Conclusions:**

These studies suggest that inhibitory phosphorylation of RLC is not a substantial regulatory mechanism, although we cannot rule out its role in other cellular processes or perhaps other types of cells or tissues in vivo.

## Background

Non-muscle myosin II is expressed in nearly every eukaryotic cell, where it plays critical roles in a number of cellular processes, including cell division and cell migration. Myosin II molecules are comprised of two heavy chains (MHC), two essential light chains (ELC) and two regulatory light chains (RLC). The MHC consists of a globular head domain that contains that actin binding and ATPase properties, a linker region that contains the binding sites for the ELC and RLC and a coiled-coil rod domain that allows the MHC to dimerize and assemble into bipolar filaments. Myosin II is in constant equilibrium between monomeric and filamentous forms. The cell achieves spatio-temporal control of myosin II assembly and activation by modulation of this equilibrium, primarily through phosphorylation events.

There are two groups of residues on the RLC that are phosphorylated by distinct kinases and have contrasting effects on myosin II biophysical properties. The first group is Thr18/Ser19. These residues are phosphorylated by myosin light chain kinase, Rho kinase and others [1]. Phosphorylation at Thr18/Ser19 is a well-established regulatory mechanisms that increases the actin-activated ATPase activity of the holoenzyme and shifts the molecule into a filamentous state [2,3]. Therefore, Thr18/Ser19 phosphorylation essentially "activates" the myosin molecule to produce force. The second group of phosphorylated residues is at the N-terminus of the RLC at Ser1, Ser2 and Thr9 [4]. These residues have been shown to be phosphorylated by PKC [5]. Biophysical studies showed that PKC phosphorylation leads to a 9-fold increase in the Km of MLCK for RLC, thereby indirectly favoring a less active state for the myosin II itself [6]. Further in vitro studies with Xenopus myosin II using alanine substitution at either Ser1/Ser2 or Thr9 followed by PKC pre-phosphorylation of the remaining non-mutated residue identified Thr9 as the critical inhibitory phosphorylation event [7].

Live cell studies showed that phosphorylation at Ser1/Ser2 (but not Thr9) is elevated 6-12 fold higher in cells arrested in mitosis versus non-mitotic cells [8]. Release of the cells from mitotic arrest results in a decrease in Ser1/Ser2 phosphorylation over the next hour, as the cells progress through cell division [8]. These studies support the hypothesis that "inhibitory" phosphorylation at Ser1/Ser2, and perhaps Thr9, is a mechanism by which the contractile machinery for cell division is held in an inactive form during metaphase then activated after the metaphase/anaphase transition. One recent study identified elevated Ser1 phosphorylation in fibroblasts following treatment with platelet-derived growth factor (PDGF) [9], concordant with disassembly of acto-myosin stress fibers. Based on visual scoring, stress fiber disassembly was reported to be attenuated with expression of an un-phosphorylatable RLC at Ser1/Ser2 [9]. However, aside from this single report, no studies have addressed the importance of Ser1/Ser2/Thr9 phosphorylation in live cell settings.

The goal of our studies was to quantify the effect of RLC inhibitory phosphorylation at Ser1/Ser2/Thr9 on myosin II activity and assembly in live cells in multiple settings. Utilizing phospho-specific antibodies and GFP-tagged RLC constructs with mutations at the putative activating and inhibitory sites, we find clear modulatory roles for Thr18/Ser19 phosphorylation. However, our data is inconsistent with the hypothesis that Ser1/Ser2/Thr9 phosphorylation significantly regulates myosin II function in live cells in the settings of cytokinesis or cell spreading.

## Results and Discussion

**Figure 1:**
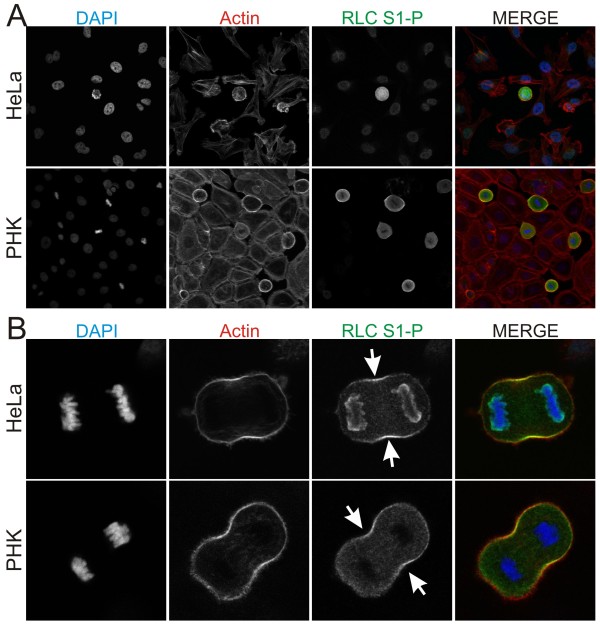
**RLC Ser1 phosphorylation is elevated in mitotic cells and localizes to the contractile ring**. (A) HeLa and primary human keratinocytes (PHK) under normal growth conditions were fixed and immunostained for RLC Ser1-P (green) and stained for actin (red). (B) RLC Ser1-P is specifically enhanced in the contractile ring of dividing cells.

Using a phospho-specific antibody to RLC Ser1, we immunostained HeLa and primary human keratinoctyes under normal growth conditions. In agreement with published reports [8,10], RLC Ser1 phosphorylation is dramatically enhanced in mitotic cells (Figure 1A). The previous study by Yamakita and colleagues examined RLC phosphorylation following a semi-synchronous release from mitotic arrest in metaphase. In doing so they observed that Ser1/Ser2 phosphorylation decreased as the cells progressed through mitosis. However, it is not clear if the residual Ser1/Ser2 phosphorylation in their "released" cells was in cells that had not yet transitioned in to anaphase or in cells that were in anaphase. To determine if RLC remains phosphorylated at Ser1 in cells that are actively dividing, we imaged HeLa cells in the anaphase state. In doing so, we observed that RLC Ser1 phosphorylation is enhanced specifically in the contractile ring of anaphase cells (Figure 1B). This was also true in primary human keratinocytes (Figure 1B, bottom row). The Ser1-P antibody also strongly localizes to peri-chromosomal regions in the HeLa cell line but not the primary keratinocytes. As we never observe endogenous RLC or MHC localizing to these areas (data not shown) this peri-chromosomal staining either represents a very small percentage of the total myosin II in the cell or it represents spurious antibody reactivity with something other than the RLC.

It appears contradictory that myosin II is phosphorylated at putative inhibitory sites in the contractile ring, where myosin II activity is proposed to be the dominant force contributing to furrow ingression. However, inhibitory phosphorylation and activated myosin II in a specific local, such as the contractile ring, may not be mutually exclusive. In *Dictyostelium discoideum*, myosin II disassembly is necessary for efficient completion of cytokinesis [11]. Similarly, it may be that in mammalian cells, RLC inhibitory phosphorylation contributes to disassembly of myosin II in the contractile ring as the furrow ingresses.

**Figure 2:**
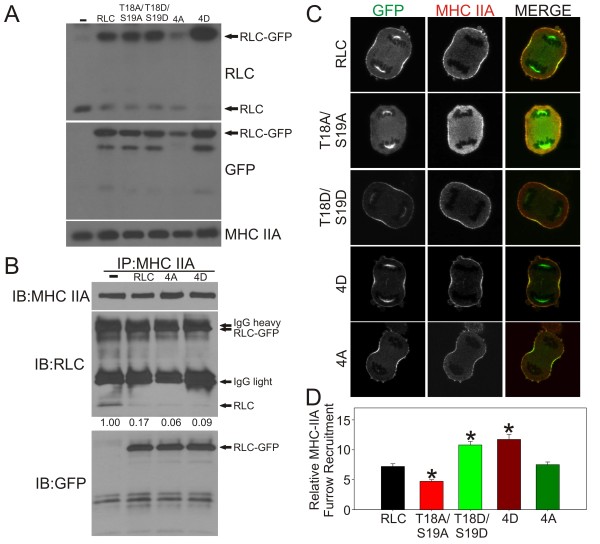
**Expression, localization and assembly of MHC IIA during cell division**. (A) Whole cell lysates of HeLa cells expressing the indicated RLC construct were probed for total RLC, GFP and MHC IIA. (B) MHC IIA was immunoprecipitated from the indicated cell line and the eluate was probed for MHC IIA, RLC and GFP. Note that the RLC-GFP and IgG heavy chain migrate too close to differentiate. The number under each construct indicates the relative amount of RLC present after normalization to MHC IIA based on densitometry. (C) Anaphase cells expressing the indicated RLC construct (green) were fixed and stained for MHC IIA (red). Representative images are shown. (C) MHC IIA recruitment was quantitated for a minimum of 13 cells. Data are mean +/- SEM. * indicates p = < 0.003.

To determine if modulation of the putative inhibitory residues would alter myosin II behavior during cell division, we created GFP-tagged RLC constructs with non-phosphorylatable alanine substitutions (RLC-4A) and phosphomimetic aspartic acid substitutions (RLC-4D) at Ser1/Ser2/Thr9/Thr10. Thr10 was previously shown to be a secondary phosphorylation site when Thr9 is mutated [7], so this residue was also mutated in all constructs. As controls, we also created non-phosphorylatable (RLC-T18A/S19A) and phosphomimetic (RLC-T18D/S19D) mutations at the activation sites. These constructs, along with the wild type RLC-GFP, were stably transfected into tetracycline-responsive HeLa cells, allowing temporally regulated expression of the mutated RLC-GFP proteins to avoid any chance of compensatory background changes or adaptation in cells carrying mutant constructs. When induced, these constructs expressed at slightly higher levels than the endogenous RLC and oftentimes appear to suppress expression of the endogenous RLC (Figure 2A), presumably resulting in a higher replacement of exogenous RLC on the myosin II molecule. Note that the RLC antibody used appears to have a lower affinity for the RLC-4A construct. To further quantitate the percent replacement of RLC-GFP for endogenous RLC we performed immunoprecipitation of MHC IIA followed by western blotting (Figure 2B). Unfortunately, the IgG heavy chain co-migrates with the RLC-GFP constructs, preventing any ratio analysis of RLC:RLC-GFP. Use of a mouse primary antibody for the IP followed by a rabbit antibody for western blotting or use of immuno-depleted secondary antibodies did not alleviate this problem. However, we were able to ratio the endogenous RLC to the amount of MHC IIA immunoprecipitated. Under the assumption that the remaining MHC IIA is occupied with RLC-GFP, we estimate our percent replacement to be ~80-95%.

To determine if mutations at the putative inhibitory sites altered the ability of myosin II to localize to and assemble in the furrow during cell division, we stained HeLa cells for the most abundant MHC isoform, MHC IIA. Recruitment of myosin II to the furrow was assessed by fluorescent microscopy (Figure 2C), and quantitated by normalizing peak cortical furrow signal to cytoplasmic signal in a number of cells (Figure 2D). Relative to expression of wild type RLC, expression of RLC-T18A/S19A resulted in a decrease in the amount of cortically assembled myosin II in the furrow (Figure 2C &2D). In contrast, expression of RLC-T18D/S19D resulted in a relative overassembly of myosin II in the furrow (Figure 2C &2D). If phosphorylation of RLC at Ser1/Ser2/Thr9 inhibits myosin II assembly during cytokinesis, then expression of RLC-4D should result in a decrease in myosin II cortical signal in the furrow. This does not appear to be the case (Figure 2C &2D). In fact furrow recruitment was slightly elevated in this setting. Furthermore, myosin II cortical assembly in RLC-4A expressing cells was statistically indistinguishable from wild type RLC expressing cells. This data suggests that phosphorylation of Ser1/Ser2/Thr9 does not alter myosin II assembly levels in the contractile ring.

Note that the RLC-GFP constructs often localize to the spindle independent of the MHC (Figure 2C). We do not see this spindle localization when we immunostain for endogenous RLC protein (data not shown). This spindle localization appears to be different than the peri-chromosomal staining seen with the Ser1-P antibody (Figure 1B). Therefore, these appear to be independent anomalies from as of yet unknown origins.

**Figure 3:**
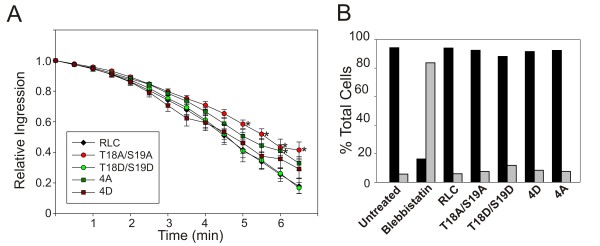
**Analysis of furrow ingression and multinucleation**. (A) HeLa cells expressing the indicated RLC construct were imaged throughout cell division. Furrow width was measured every 30 seconds for a minimum of 5 cells. Data are mean +/- SEM. * indicates p = <0.05. (B) HeLa cells expressing the indicated RLC construct or treated with 60 uM blebbistatin for 24 hours were analyzed for nucleation state using propidium iodide and flow cytometry. Black bars represent cells with DNA content of less than 4*n*. Grey bars represent cells with DNA content of 4*n *or greater.

It remains possible that phosphorylation at Ser1/Ser2/Thr9 may alter myosin II activity during cell division, resulting in an altered rate of furrow ingression. To investigate this, we measured furrow ingression rates of dividing cells expressing each of the RLC constructs (Figure 3A). The only cells that consistently had a statistically significant slower furrow rate relative to wild type were the cells expressing RLC-T18A/S19A. Surprisingly, even these cells were able to complete furrow ingression and divide successfully, similar to previous reports [12]. Furthermore, analysis of nucleation state as an indicator of multinucleation revealed that none of the RLC mutant constructs induced a multinucleation phenotype (Figure 3B). The myosin II inhibitor blebbistatin did cause a significant shift into a multinucleated state, indicating an absolute necessity for myosin II activity (Figure 3B). Similar results were obtained in HEK293 cells (data not shown). In summary, our data suggests that modulation of the RLC activating sites (Thr18/Ser19) clearly impacts myosin II recruitment to the furrow, but has modest effects on furrow rates, and no detectable effect on completion of furrowing as assessed by multinucleation. Phosphomimetic substitution of the putative inhibitory sites (Ser1/Ser2/Thr9) on RLC conferred an effect on assembly opposite of what would be predicted if these sites are inhibitory and had little or no effect on furrow rates. Alanine substitutions at the putative inhibitory sites conferred a phenotype virtually indistinguishable from wild type.

**Figure 4:**
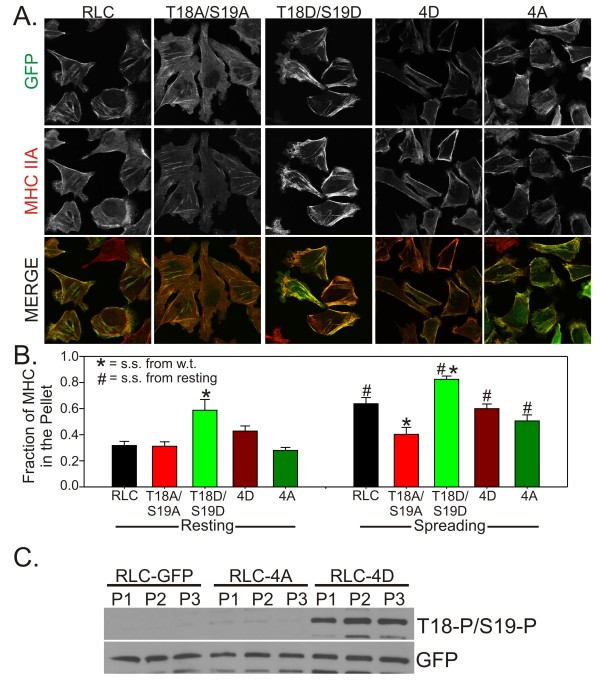
**MHC IIA assembly in resting and spreading cells**. (A) HeLa cells in normal growth conditions expressing the indicated RLC-GFP construct (green) were fixed and immunostained for MHC IIA (red). (B) Triton-insoluble fractionation was performed on HeLa cells in normal growth conditions ("resting") or actively spreading for 1 hr. Data are mean +/- SEM. N ≥ 6 for resting and N ≥ 9 for spreading. # indicates p = < 0.05 relative to the same cells in resting conditions. * indicates p = < 0.05 relative to the wild type RLC in the same conditions. (C) Multiple pellet (P) fractions from the triton-insoluble fractionation studies were assayed for their Thr18/Ser19 phosphorylation levels. GFP was used to show that each pellet contains similar amounts of the indicated RLC-GFP construct.

We next sought to determine if modulation of RLC Ser1/Ser2/Thr9 phosphorylation could affect myosin II assembly in non-mitotic cells. HeLa cells under normal growth conditions that were expressing the RLC constructs were immunostained for MHC IIA (Figure 4A). Relative to cells expressing wild type RLC, cells expressing RLC-T18A/S19A displayed fewer stress fibers and generally appeared larger, indicating a possible defect in myosin II assembly and contractility (Figure 4A, column 1&2). In contrast, cells expressing RLC-T18D/S19D appeared to have enhanced myosin II assembly in stress fibers (Figure 4A, column 3). MHC IIA assembly in cells expressing RLC-4D or RLC-4A, which might be predicted to cause an under-assembly or over-assembly defect, respectively, was indistinguishable from the wild type (Figure 4A, columns 4&5).

To quantitate assembly levels of myosin II when different RLC constructs were expressed, we used triton-insoluble fractionation to separate cytoskeletal and cytosolic cellular fractions. Expression of RLC-T18D/S19D resulted in an increase in assembled myosin II (Figure 4B). Expression of RLC-T18A/S19A, RLC-4A and RLC-4D did not significantly alter resting assembly levels relative to wild type RLC (Figure 4B). To determine if forcing the cells to disassemble and reassemble their cytoskeleton might reveal a role for RLC Ser1/Ser2/Thr9 phosphorylation, we trypsin-collected the cells and re-plated on fibronectin coated dishes for 1 hour before performing triton-insoluble fractionation. Consistent with RLC phosphorylation increasing at Thr18/Ser19 during cell spreading [13], we observed that in cells expressing wild type RLC-GFP, myosin II assembly levels increased during cell spreading (Figure 4B, compare RLC in resting versus spreading). Expression of RLC-T18A/S19A resulted in myosin II that fails to significantly increase assembly level during spreading and remained at a similar assembly level to non-spreading cells (Figure 4B). These cells often show aberrant morphology during spreading (data not shown), suggesting that proper myosin II activation and/or assembly are needed for proper cell spreading. Myosin II in cells expressing RLC-T18D/S19D remains at elevated assembly levels during spreading (Figure 4B). Therefore, substitution of unphosphorylatable or phosphomimetic residues at the T18/S19 activating sites cause myosin II to under-assemble or over-assemble, respectively, during cell spreading. This is consistent with Thr18/Ser19 phosphorylation being a dominant mechanism regulating myosin II assembly. In contrast, in cells expressing RLC-4A or RLC-4D, myosin II assembly levels increased during spreading similar to the behavior of wild type RLC (Figure 4B). This data is inconsistent with RLC Ser1/Ser2/Thr9 phosphorylation significantly regulating myosin II assembly levels during cell spreading.

In multiple assays performed in this work the RLC-4D construct conferred a phenotype opposite of what would be expected by the current model. Namely, RLC-4D caused an over-assembly of MHC IIA in the furrow (Figure 2C and 2D) and RLC-4D appeared to cause a slight over-assembly of MHCIIA in resting cells (Figure 4B). To determine if Thr18/Ser19 phosphorylation was altered when RLC-4D was expressed we performed western blotting on the cytoskeletal fractions used for analysis in Figure 4B. Relative to both the wild type RLC-GFP and RLC-4A constructs, expression of RLC-4D caused a substantial increase in Thr18/Ser19 phosphorylation (Figure 4C). This result is once again inconsistent with the model that Ser1/Ser2/Thr9 phosphorylation is inhibitory towards assembly. We speculate that addition of multiple aspartate residues to the N-terminus of the RLC might be inhibiting 10S formation, possibly leaving the RLC more susceptible to the activation kinases. Alternatively, it is possible that addition of these aspartate residues inhibits interaction between the RLC and potential phosphatases. Clearly, further studies are needed to resolve this unexpected result.

Recent work by the Sellers lab has shown that despite some minor differences, heavy meromyosin (HMM) molecules with GFP-tagged RLC constructs can be regulated via phosphorylation in a similar manner to myosin molecules with untagged RLC [14]. Although this work by Kengyel and colleagues analyzed an N-terminal GFP fusion (in contrast to our C-terminal GFP fusion), their studies show that a GFP-tagged RLC can associate with myosin heavy chains and behave relatively normal. Additionally, our studies show that cells expressing the wild type RLC-GFP behave normal during cytokinesis (Figure 3) and during adhesion-stimulated filament assembly (Figure 4B). In conjunction with our IP data (Figure 2B) suggesting a substantial replacement of the endogenous RLC on the myosin II population, this data argues strongly for both normal association and function for the RLC-GFP used in these studies.

Due to the remaining presence of a small level of endogenous RLC in these cells, we cannot entirely rule out a role for RLC Ser1/Ser2/Thr9 phosphorylation in regulating myosin II in our system. However, it is important to note that each of the assays performed here, with the exception of assessing a failure to complete cytokinesis by measuring nucleation state, demonstrate that point mutations at Thr18/Ser19 significantly alter myosin II function in live cells. Therefore, the RLC-T18/S19 constructs help to establish each assay as a valid method to investigate the magnitude of the role of Ser1/Ser2/Thr9 phosphorylation. Collectively, these assays show that even in the presence of a small amount of endogenous RLC, RLC-T18/S19 mutants display phenotypes congruous with the established model, while RLC-4A and RLC-4D mutants display phenotypes similar to the wild type RLC. To completely eliminate the possibility that residual RLC is masking an effect of Ser1/Ser2/Thr9 phosphorylation, future experiments might utilize siRNA/shRNA approaches in conjunction with resistant RLC constructs. This may help to more conclusively determine any role for RLC Ser1/Ser2/Thr9 phosphorylation in live cells.

It is intriguing that expression of RLC-T18A/S19A delayed furrow ingression but did not inhibit cytokinesis in two different cell types. Again, it is possible that the introduced RLC-GFP constructs are not fully displacing the endogenous RLC from the MHC and therefore, the small amount of residual wild type myosin II is enough to drive division. Alternatively, it is possible that basal levels of myosin II ATPase, even in the absence of Thr18/Ser19 phosphorylation, are sufficient to drive furrow contraction. Future experiments may differentiate between these possibilities.

In addition to MLCK, RLC can also be phosphorylated at Thr18/Ser19 by a number of other kinases, including citron kinase [15], Rho-kinase [16], ZIPkinase [17] and others. However, the only kinase tested for its ability to interact with and phosphorylate RLC following PKC phosphorylation was MLCK. Also, the dominant myosin phosphatase at Thr18/Ser19 in most cells consists of PP1cδ and myosin phosphatases target subunit (MYPT) [18]. This phosphatase has also not been tested for its ability to interact with and dephosphorylate RLC following PKC phosphorylation. Therefore, RLC phosphorylation at Thr18/Ser19 is controlled by a number of different regulators, but only one of those, MLCK, has been tested for its efficacy following Ser1/Ser2/Thr9 phosphorylation. It is possible that the ability of the other kinases or phosphatase to phosphorylate or dephosphorylate RLC at Thr18/Ser19 is not affected by Ser1/Ser2/Thr9 phosphorylation. If this scenario were true then it would prescribe two additional possibilities. First, the activity of the other kinases or inhibition of the phosphatase may be compensating for abrogated MLCK activity following Ser1/Ser2/Thr9 phosphorylation in live cells. Second, processes that are highly dependent on MLCK phosphorylation of RLC may be more susceptible to modulation by Ser1/Ser2/Thr9 phosphorylation. Future experiments should investigate the effect of Ser1/Ser2/Thr9 phosphorylation on the ability of other regulators of myosin II to interact with and modulate myosin II activity. Additionally, future experiments investigating alternative cell types or alternative cellular processes, especially those that are MLCK dependent, may yet unveil a more significant role for Ser1/Ser2/Thr9 phosphorylation in live cells.

## Conclusions

The data presented here argues that RLC Ser1/Ser2/Thr9 phosphorylation does not significantly regulate myosin II assembly during cytokinesis and cell spreading in HeLa cells. These studies further reinforce the importance of RLC Thr18/Ser19 phosphorylation on myosin II assembly and suggest that alternative mechanisms of disassembling myosin, such as RLC Thr18/Ser19 de-phosphorylation, MHC phosphorylation [19,20] and Mts1 binding [21], are more relevant in live cells.

## Methods

### Cell Culture

HeLa Tet-OFF were a kind gift of Bob Adelstein and were maintained in MEM with 10% fetal bovine serum and 1% penicillin/streptomycin. Polyclonal stable cell lines were created by transfecting 1 ug plasmid DNA into HeLa Tet-OFF using the Lonza Nucleofector (solution kit R, program I-13). Cells were selected in 300 mg/ml hygromycin and maintained in 1 ug/ml doxycycline. Approximately 20-100 colonies were pooled for each cell line. To identify responsive cells, Doxycycline (Sigma) was removed from the media for 2-3 days and GFP positive cells were isolated using flow cytometry. These cells were immediately placed back in doxycycline and stock cultures were maintained in Doxycycline throughout culturing. Cells were cultured in doxycycline-free media for 2-4 days prior to performing experiments. Primary human keratinocytes were isolated from discarded, de-identified human neonatal foreskins as previously described [22]. These were obtained from University Hospitals Rainbow Babies and Children's Hospital (Cleveland, OH) following approval from the University Hospitals Internal Review Board (material transfer #UNI-37894). The keratinocytes were maintained in keratinocyte serum-free medium (Gibco, Invitrogen Cell Culture).

### Creation of RLC mutants

The plasmid pTRE-GFP was a kind gift of Bob Adelstein and was created as previously described [23]. pTRE-GFP was modified to introduce a BamH1 restriction site immediately upstream of Age1 to create the vector pTRE3-GFP. MRLC2 cDNA (gene MYL12B; gene ID 103910) was purchased from ATCC. BamH1 and Age1 restriction sites were introduced at the 5' end and 3' end, respectively, of the RLC cDNA using PCR. This PCR product was then ligated into the pTRE3-GFP vector using the BamH1 and Age1 sites to create pTRE3-RLC-GFP that expresses the RLC protein with the EGFP reporter at the C-terminal end. pTRE3-RLC-GFP-4A and pTRE3-RLC-GFP-4D constructs were created using standard PCR mutagenesis techniques followed by ligation into pTRE3-GFP using the BamH1 and Age1 sites. For RLC-T18A/S19A and RLC-T18D/S19D, dsDNA oligonucleotides were purchased from DNA2.0 with the desired point mutations, a 5' BamH1 site and 3' Xma1 site. These were then digested and ligated into pTre3-RLC-GFP using BamH1 and Xma1 sites to create pTRE3-RLC-GFP-T18A/S19A and pTRE3-RLC-GFP-T18D/S19D. All DNA segments subjected to PCR were sequenced to confirm absence of PCR-generated errors.

### Western Blotting and Immunoprecipitation

For western blotting the following antibodies were used: GFP, (Invitrogen catalog #A6455), MHC IIA (Biomedical Technologies catalog #BT-567), RLC (Proteintech Group catalog #10324-1-AP), RLC T18-P/S19-P (Cell Signaling catalog #3674S).

For immunoprecipitation myosin IIA antibody (Abcam; catalog #A55456) was pre-incubated with ProteinG Dynabeads (catalog #100.03D). Cells were lysed in lysis buffer (20 mM Tris pH 7.5, 1% Triton X-100, 10% glycerol, 250 mM NaCl, 2 mM EDTA, 25 mM β-glycerophosphate, phosphatase inhibitor cocktail 2 and 3 (Sigma; catalog #P5726 and #P0044) and protease inhibitor cocktail (Sigma; catalog #P8340). The lysate was then incubated with the Dynabead-ProteinG/antibody complex. Following 3 washes in lysis buffer, the bound protein was eluted directly in 2× sample buffer supplemented with protease and phosphatase inhibitor cocktails.

### Immunofluorescence

Fixation and immunostaining was performed as previously described [24], except that primary antibodies were incubated overnight at 4°C. The following antibodies were used: GFP (Invitrogen, A-6455), RLC (Proteintech Group, 10324-1-AP), RLC S1-P (ECM Biosciences, MP3461), MHC IIA (Sigma, M8064). Samples were mounted in Vectashield (Vector Laboratories, H-1200). Images were acquired on a Leica TCS-SP2 with a plan-apochromat 63x/1.4 NA oil objective.

### Myosin II furrow recruitment

Following fixation and immunostaining for MHC IIA, a single confocal image was taken of anaphase cells at a z-position corresponding to their greatest length. Using ImageJ, a single 10 pixel-wide line was drawn through the contractile ring spanning both sides of the cell. Cortical myosin II was defined as the mean of the two peak intensities in the line histogram. Cytoplasmic myosin II was defined as a mean of the intensities between those peaks. The cortical to cytoplasmic ratio was used for comparative analysis.

### Furrow ingression

Mitotic cells were collected using the shake-off method. These cells were replated on tissue culture dishes pretreated with 20 ug/ml fibronectin (Sigma-Aldrich, F1141). Images were collected every 15 seconds using a Leica DMIRE2 with a plan 20x/0.4 NA objective. Furrow to furrow distance was measured using the line draw tool in ImageJ.

### Nucleation state analysis

Cells were analyzed for their nucleation state using propidium iodide as previously described [24]. Only GFP-positive cells were analyzed.

### Triton-insoluble fractionation

Cells under normal growth conditions or actively spreading for 1 hr on fibronectin coated tissue culture dishes were lysed in TIF buffer (50 mM Tris-HCl, pH 7.4, 5 mM NaCl, 140 mM potassium-acetate, 0.6% Triton X-100, 1 mM EDTA, 5 mM EGTA, 5 mM DTT and protease/phosphatases inhibitors). Soluble and insoluble fractions were separated by centrifugation at 8,000 × *g *for 15 min. The soluble supernatant was removed and combined with an equal volume 2× Laemmli sample buffer. The pellet was resuspended in the same amount of 2× Laemmli sample buffer as the supernatant, followed by dilution with an equivalent amount of TIF buffer. Samples were boiled for 3 minutes and sonicated briefly prior to being subject to western blot analysis. Western blot band intensities were quantitated using ImageJ.

## Authors' contributions

JB participated in design of the study, participated in all data acquisition and drafted the manuscript. LL participated in the triton-insoluble fractionation studies. JC participated in the keratinocyte studies. TE conceived of the study, and participated in its design and coordination and helped to draft the manuscript. All authors read and approved the final manuscript.
