# Correction: Analysis of the Role of Ser1/Ser2/Thr9 Phosphorylation on Myosin II Assembly and Function in Live Cells

**DOI:** 10.1186/1471-2121-13-11

**Published:** 2012-04-18

**Authors:** Jordan R Beach, Lucila S Licate, James F Crish, Thomas T Egelhoff

**Affiliations:** 1 Department of Cell Biology, Lerner Research Institute NC-10, Cleveland Clinic, 9500 Euclid Avenue, Cleveland, OH 44195, USA; 2 Department of Physiology and Biophysics, Case Western Reserve University, 10900 Euclid Avenue, Cleveland, OH 44106, USA

## Methods

In the Methods section of our original manuscript [1] under the subheading “Creation of RLC mutants” the third sentence reads “MRLC2 cDNA (gene MYL12B; gene ID 103910) was purchased from ATCC”. This sentence is incorrect. The manuscript should read “MRLC1 cDNA (gene MYL9; gene ID 10398) was purchased from ATCC”. This correction does not in any way change the conclusions or any of the data in the manuscript. These two redundant genes produce essentially identical proteins, we simply listed the wrong one in the methods section.
